# P-1920. Impact of Vancomycin AUC-based Dosing in a Long-term Acute Care Hospital System

**DOI:** 10.1093/ofid/ofaf695.2089

**Published:** 2026-01-11

**Authors:** Katherine M Shea, Shelby Hillery, Dylan J LeBlanc, Athena L V Hobbs

**Affiliations:** Cardinal Health, Austin, TX; Cardinal Health, Austin, TX; Cardinal Health, Austin, TX; Cardinal Health, Austin, TX

## Abstract

**Background:**

Vancomycin therapeutic monitoring guidelines were published in March 2020 and indicated that for serious infections due to methicillin-resistant Staphylococcus aureus, a change from using surrogate troughs of 15 to 20mg/L to calculation of an area under the curve (AUC) over 24 hours to minimum inhibitory concentration (MIC) of 400-600 mg*h/L was recommended. Literature regarding the impact of AUC-based dosing within the post-acute setting is lacking. As of 2021, trough-based dosing was the standard of care within a long-term acute care hospital (LTACH) system. The system antibiotic stewardship committee implemented AUC-based dosing using a pharmacokinetic calculator as the standard of care. Investigators sought to assess the impact of vancomycin AUC-based dosing.

**Methods:**

This was a retrospective analysis within 6 LTACHs comparing the incidence of nephrotoxicity before (01/2022-12/2022) and after (01/2024-12/2024) implementation of vancomycin AUC-based dosing and monitoring. Nephrotoxicity was defined as a serum creatinine 1.5 times the patient’s baseline and occurred during or within 3 days after vancomycin discontinuation or patient discharge, whichever came first. Patients receiving vancomycin for greater than equal to 2 days within the study period were included. Those with end-stage renal disease or a baseline serum creatinine of greater than 2 mg/dL were excluded. Patient clinical data, including demographics, comorbidities, concomitant nephrotoxins, medication administration records, and laboratory values were obtained from the medical record. Chi-square was used for nominal data and a 2-sided t-test for continuous data.

**Results:**

There were 80 patients included in the analysis, 42 in the trough-based pre-group and 38 in the AUC-based post-group. The majority of patients were male (57.5%), and the mean age was 67.9 (SD, 15.2 years). The mean duration of vancomycin was 12.0 (SD, 8.4 days). 58.8% of patients received at least 1 concomitant nephrotoxic medication. The post-implementation group experienced a non-significant reduction in nephrotoxicity incidence (28.9 vs. 50%; p=0.055).

**Conclusion:**

Implementation of vancomycin AUC-based dosing and monitoring in an LTACH system was associated with a non-significant reduction in nephrotoxicity.

**Disclosures:**

Katherine M. Shea, PharmD, BCIDP, Cardinal Health: Employee|Cardinal Health: Stocks/Bonds (Public Company) Shelby Hillery, Pharm.D, Cardinal Health: Employee Dylan J. LeBlanc, Pharm.D, BCPS, Cardinal Health: Employee Athena L.V. Hobbs, Pharm.D., BCIDP, FIDSA, Cardinal Health: Employee

